# Recapture Heterogeneity in Cliff Swallows: Increased Exposure to Mist Nets Leads to Net Avoidance

**DOI:** 10.1371/journal.pone.0058092

**Published:** 2013-03-05

**Authors:** Erin A. Roche, Charles R. Brown, Mary Bomberger Brown, Kristen M. Lear

**Affiliations:** Department of Biological Sciences, University of Tulsa, Tulsa, Oklahoma, United States of America; University of Debrecen, Hungary

## Abstract

Ecologists often use mark-recapture to estimate demographic variables such as abundance, growth rate, or survival for samples of wild animal populations. A common assumption underlying mark-recapture is that all animals have an equal probability of detection, and failure to meet or correct for this assumption–as when certain members of the population are either easier or more difficult to capture than other animals–can lead to biased and inaccurate demographic estimates. We built within-year and among-years Cormack-Jolly-Seber recaptures-only models to identify causes of capture heterogeneity for a population of colonially nesting cliff swallows (*Petrochelidon pyrrhonota*) caught using mist-netting as a part of a 20-year mark-recapture study in southwestern Nebraska, U.S.A. Daily detection of cliff swallows caught in stationary mist nets at their colony sites declined as the birds got older and as the frequency of netting at a site within a season increased. Experienced birds’ avoidance of the net could be countered by sudden disturbances that startled them into a net, such as when we dropped a net over the side of a bridge or flushed nesting cliff swallows into a stationary net positioned at a colony entrance. Our results support the widely held, but seldom tested, belief that birds learn to avoid stationary mist nets over time, but also show that modifications of traditional field methods can reduce this source of recapture heterogeneity.

## Introduction

The study of wild animal populations often requires that ecologists base their inferences on a sample of the population that can be uniquely marked and followed. In this process, known as mark-recapture, the proportion of animals originally caught and uniquely marked that are subsequently re-caught is used to generate demographic estimates of abundance, growth rate, or survival that may be applied to the entire population [1]. Because this general methodology relies so heavily on the ratio of marked animals that are re-encountered to unmarked animals, one of the most important assumptions is that all animals within the population or within subsets of the population being compared have an equal probability of capture or detection [1]. However, it is well known that capture and marking can alter individuals’ behavior, making them either more or less likely to be recaptured [2]–[9].

Acknowledged sources of variation in recapture probability generally fall into two categories, both of which may be present in any given field study [10]: (*i*) extrinsic factors such as weather [11], [12], capture site [13], capture method [14]–[19], tag loss [20], or observer-related effects [21], [22], and (*ii*) intrinsic morphological and behavioral characteristics, commonly referred to as “individual heterogeneity,” such as age [23]–[26], sex [24], [27], [28], social rank [29], social community and site fidelity [30], foraging strategy [31], body size or condition [32]–[35], time spent at a location [36], size of the study area relative to the movement of marked individuals [37], [38], or breeding stage [13], [39]–[41]. It has also been proposed that consistent individual differences in behavior (commonly referred to as “personality”) lead to capture heterogeneity [42]. If any of these possibilities apply, it may appear that sampled individuals have very different life histories relative to the total population that ecologists are interested in studying. As heterogeneity increases, so too does the bias associated with demographic estimates such as apparent survival [43]–[46]; in the case of severe capture heterogeneity, this may lead to an inaccurate inference of age effects on apparent survival [13]. A failure to accurately account for detection heterogeneity among individuals can additionally lead to flawed estimates of animal abundance [6], [45], [47], population growth and size [44], [48], [49], or species diversity [50]–[52], and may make it difficult to detect environmental drivers of demography [53], to infer the form of natural selection [2], to measure survival differences among groups of individuals [54], or to test for evidence of senescence among older animals [55], [56].

Recognition of the potentially serious consequences of failure to correct for detection heterogeneity has led to the development of quantitative methods that incorporate the complexities of recapture probability into mark-recapture models. More traditional techniques include statistically accounting for the presence of “transient” individuals, who are captured once and never again [57], as well as “trap-dependent” effects, where initial capture of an animal affects future probability of recapturing that individual [58], [59]. Closed-population models recognize heterogeneity in capture probabilities when estimating population abundance [60]–[64], and recent advances in the use of multi-event or open-population mixture models allow investigators to either specify a finite number of capture groups of varying capture probability [6]–[8] or account for random variability among individual recapture probabilities [9]. Hierarchical models can account for heterogeneity among individuals as well as variation among spatiotemporal replicates [20], [65]; these techniques may be particularly powerful when analysts are faced with sparse datasets [66].

We believe there is a need for ecologists to more closely examine how sampling methods influence the selection of subsets of a study population. When possible, the inclusion of descriptive covariates for factors believed to influence detection can help researchers adjust demographic estimates for heterogeneity while also revealing the source of the heterogeneity. By investigating interactions between specific sources of detection heterogeneity, we may be able to devise means to avoid or minimize recapture variation during field sampling. Although many studies regard recapture probability as a “nuisance” parameter to be dealt with statistically [1], differences among individuals in their response to capture may at times be biologically meaningful, particularly if they reveal how different subsets of the population learn through experience.

We explored sources of recapture heterogeneity in a long-term mark-recapture investigation of social behavior in a population of colonially nesting cliff swallows (*Petrochelidon pyrrhonota*) in southwestern Nebraska, U.S.A. [67]–[70]. Our objectives were to identify to what extent probability of detection is related to individual characteristics and netting methods. We examined whether an individual's age, sex, and frequency of exposure to netting could change its behavior, ultimately influencing its probability of being detected. In this study we focus on mist nets as a capture method. Mist nets are used extensively in investigating avian demography and are commonly employed in long-term bird monitoring programs [71]–[75]. The conventional (but largely untested) wisdom is that as the frequency of mist-net operation increases at a site, birds there begin to exhibit net avoidance [76], [77].

## Materials and Methods

### Ethics Statement

This work was approved by a series of Institutional Animal Care and Use Committees of Yale University, the University of Tulsa, and the University of Nebraska-Lincoln, most recently under protocol TU-0020. Birds were captured and banded under United States Fish and Wildlife Service banding permit 20948 and a series of scientific permits issued by the Nebraska Game and Parks Commission.

### Study Animal

The cliff swallow is a colonial, insectivorous, 20–25 g passerine bird that breeds throughout western North America, building gourd-shaped mud nests underneath rocky ledges on the walls of cliffs, beneath the eaves of buildings or bridges, or inside highway culverts. The nests tend to be stacked together closely, often sharing walls [67], [78]. Cliff swallows winter in southern South America, begin arriving in our Nebraska study area in late April or early May, generally raise only one brood, and depart on fall migration by late July [67].

### Study Site

Our study area included cliff swallow colonies located along the North and South Platte rivers centered at the Cedar Point Biological Station *(*41°12.591' N, 101°38.969' W) near Ogallala, in Keith County, southwestern Nebraska, and also included portions of Garden, Deuel, Lincoln, and Morrill counties. Colonies were situated on bridges, inside culverts underneath highways or railroad tracks, underneath the eaves of buildings, and on the sides of cliffs along the shore of Lake McConaughy. Groups of nesting swallows using the same site and exhibiting at least occasional interactions were considered the same colony [67]. About 220 swallow colony sites have been monitored within the study area since 1982. The study area and colony sites are described in detail by Brown and Brown [67] and Brown [79].

### Field Methods

Beginning in 1991, we monitored the settlement of breeding cliff swallows at 25–40 colony sites each year through systematic mist-netting at each colony [68], [70]. These sites included all that were accessible for netting within an ∼10-km radius of the Cedar Point Biological Station, plus additional sites farther away within the study area which were sampled because they added to the range of colony sizes studied. Although mist-netting of swallows began in 1982, capture efforts were sporadic prior to 1991, and colony sites used in this study were restricted to those active during 1991–2010. However, birds marked prior to 1991 were included if they were re-captured at breeding colonies during 1991–2010.

**Figure 1:**
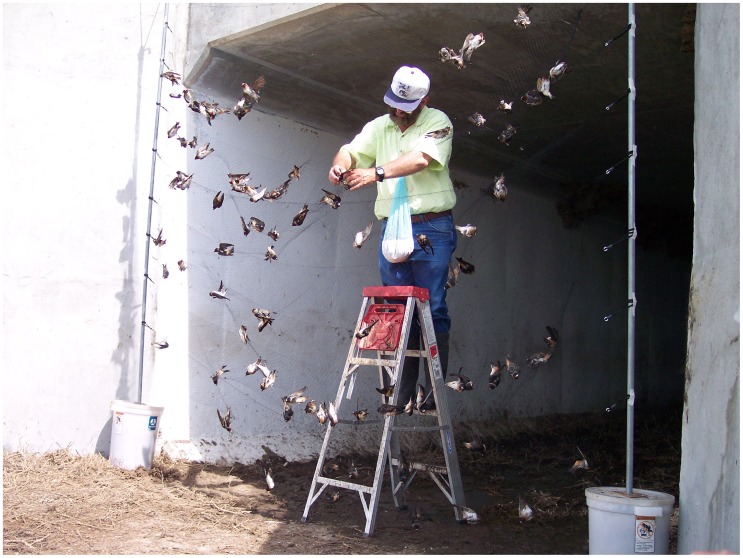
Example of a set net erected at one end of a culvert containing a cliff swallow colony (Junkyard), following a flush of birds into the net.

We used two types of mist-netting depending on the configuration of a given swallow colony. For colonies situated above dry ground or shallow water (e. g. highway or railroad culverts), we used “set” netting, in which we placed a mist net at an upwind colony entrance (Figure 1) or along the side of the bridge, and captured birds as they exited their nests into the wind and flew into the mist net. In set-netting, we erected the net only once each day, generally netted for either an entire day or a half day, and removed birds from the net continually as they were captured (requiring frequent human presence at the net and periodic disturbance to the colony). At two of the set-net colony sites (Whitetail and Junkyard) we would also occasionally perform flushes, in which a researcher would first conceal himself or herself near the downwind end of the colony and then suddenly walk or run to the other end in an attempt to direct swallows towards the colony entrance obstructed by the mist net. Birds generally flew out of their nests in large numbers in the opposite direction of the researcher, towards the net. The large numbers of birds flying within the confines of a relatively small culvert and their reluctance to collide with other flying birds meant that many individuals could not take evasive action to avoid the net and thus were caught (Figure 1). We conducted and recorded flushes at Junkyard from 2008–2010; at Whitetail, although flushing was done periodically prior to 2008, we did not begin documenting its use until 2008. Flushing was not done at Aquaduct, the third set-net site, as the nests were too high above the ground for flushing to be effective.

**Figure 2:**
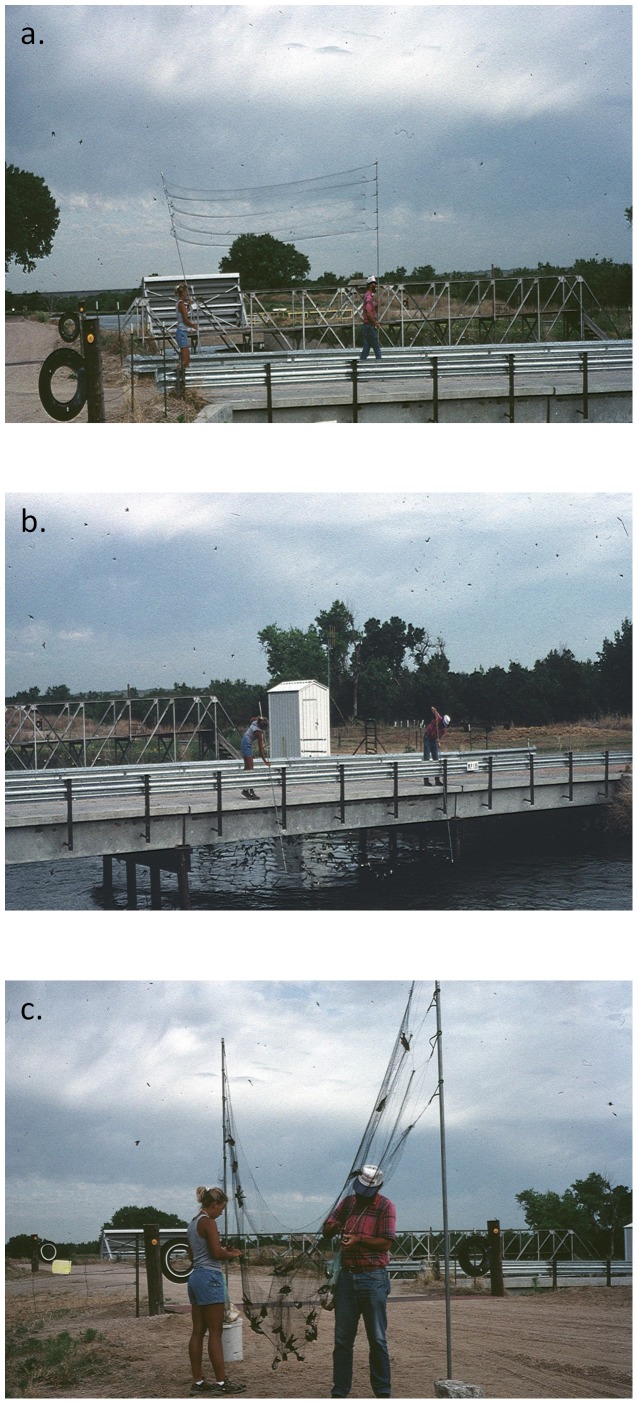
Illustration of the drop-netting method for catching cliff swallows at a bridge colony: (a) the net is carried onto the top of the bridge, (b) dropped over the side to capture birds, and (c) carried off the bridge for processing of birds. The colony pictured is Beckius. A video illustrating drop-netting of cliff swallows at CR4 is available at http://www.youtube.com/watch?v=kyfe5Sg9t0Y.

When anchoring a mist net on the ground was not practical (usually because of high water beneath the nests), we carried a net (attached to poles) onto the bridge above the nests (Figure 2a) and “dropped” the net over the side of the bridge, capturing cliff swallows as they flew out of their nests in response to the disturbance (Figure 2b). The net was then carried off the bridge and away from the colony to remove and process birds (Figure 2c), and the colony was largely undisturbed in between net drops. Sometimes two nets were used, in which two pairs of researchers dropped one over each side simultaneously, but we did not explicitly account for the effects of using one versus two nets. We would typically drop a net between 3–10 times in a day (depending on the number of birds caught on each drop) and generally only drop-netted for 3–4 hours at a site on a given day. Unlike set-netting, which was used throughout the breeding season (mid-May through August), we typically conducted drop-netting only from mid-May to late June when cliff swallows at a colony were likely to be nest- building, laying or incubating eggs, and thus inside their nests in large numbers at a given moment. Flushing, as defined here, was not done at any of the drop-net sites because the presence of water made it impossible to approach the nests from below. We rotated among the colonies, netting several times per season at each, with the number of netting visits (regardless of capture method) generally greater at larger colonies than at smaller colonies. The capture of birds is described in detail by Brown [79] and Brown and Brown [67], [68].

Sex of birds was determined using a combination of cloacal protuberance (on males) and brood patch (on females). Because females early in the nesting season had often not yet developed brood patches, and cloacal protuberances on males were sometimes difficult to discern, we used a combination of sexings from multiple captures and presence of blue coloration on the throat (more blue on males) to achieve an accuracy of >90% on sex determinations, as described by Brown and Brown [67].

### Estimating Within-Year Detection and Apparent Survival

We used RMark [80], [81] to conduct within-year Cormack-Jolly-Seber (CJS) recaptures-only analyses on six colonies where cliff swallows had been extensively netted during 1991–2010, to identify individual and occasion-specific covariates that could influence the daily probability of capturing a bird (Table 1). These colonies included three set-net sites (Aquaduct, Junkyard, and Whitetail) and three drop-net sites (Beckius, CR2, and CR4). Sample sizes for each site are shown in Table 2. Each colony site was a unique environment that influenced our ability to catch birds there to different degrees.

**Table 1:** Definitions for covariate notations used in all Cormack-Jolly-Seber mark-recapture models for a study of recapture heterogeneity in cliff swallows.

Notation	Definition
.	null model
**Age**	continuous covariate represented by a linear trend based on relative age as measured by years carrying a band as an adult, where ‘1′ indicates either the year first banded as an adult for swallows of unknown age, or the year following the hatching year for swallows of known age (we capitalize “Age” out of mark-recapture convention, e.g. “t” vs. “T” to represent a categorical time vs. continuous linear time effect)
**colony**	covariate used when estimating within-year detection probability in the analysis of flushing and indicating whether a bird was caught at either Junkyard or Whitetail
**date**	continuous covariate representing the date on which a colony was visited
**flush**	whether or not a cliff swallow was caught on a day on which flushing was done.
**FY**	categorical covariate representing first-year survival in the among-years analysis
**g**	categorical covariate representing group in the among-years analysis (SK, known age upon first capture in set-nets; SU, unknown age upon first capture in set-nets; DU, unknown age upon first capture in drop-nets)
**net**	categorical covariate used in the among-years analysis to represent the type of net used for capture
**RE**	Intercept-only random effect included for detection probability [9]
**sex**	categorical covariate (0, male; 1, female) included in all analyses
**size**	annual covariate representing colony size (number of active nests)
**total**	annual covariate representing total number of colony visits in a year
**trap**	individual categorical covariate representing whether a cliff swallow had been captured on a previous colony visit
**visit**	continuous covariate representing the number of visits that have elapsed prior to a given capture occasion (e.g., the second day a colony was netted in a year was visit 1)
**unk**	categorical covariate representing initial status by group in the among-years analysis (for example, individuals of known age have received a “0”, while those of unknown age would have received a “1”)
**year**	survival/detection varying by year

**Table 2:** Sample sizes and Cormack-Jolly-Seber recapture-only model specifications for eight analyses of cliff swallows in a mark-recapture study in southwestern Nebraska, U.S.A., 1991–2010.

Analysis	Net type	Years	Occasions	Individuals	Effective sample size	ĉ
Among-years	Both	20	20	143454	210188	7.40
Aquaduct	Set-net	10	45	8200	10820	2.50
Junkyard	Set-net	13	76	28474	43085	1.64
Whitetail	Set-net	20	77	42922	81189	1.84
Beckius	Drop-net	10	34	3965	5553	1.52
CR2	Drop-net	15	44	5859	7856	1.57
CR4	Drop-net	12	52	6638	8894	1.38
Flush	Set-net	3	54	14476	20093	1.80

Net type indicates the style of netting used to capture cliff swallows at a given colony; Years is the number of groups in the analysis except for in the among-years analysis (which had three groups); Occasions is the number of unique dates on which captures occurred across all years except for the among-years analysis in which each year was considered an occasion; Individuals is the number of different swallows included in the analysis (i.e. sometimes the same swallow was captured in multiple years); Effective sample size is the total times swallows were captured across all occasions and groups; ĉ is the measure of overdispersion associated with the analysis and used to calculate QAIC_c_ values.

Of the set-net sites, (1) Aquaduct was a 30-m wide and 6-m tall bridge, the largest physical substrate of any colony netted at and having the lowest density of nests; (2) Junkyard was a single-tunnel railroad culvert typically containing nests in higher density than at any other site and with relatively little disturbance from humans, other than train traffic overhead; and (3) Whitetail was a double-tunnel highway culvert whose low height and small size typically concentrated birds exiting their nests into a relatively confined area, often making the birds' avoidance of the net more difficult at this site than at the other sites. Of the drop-net sites, (4) Beckius was the smallest bridge with its reduced physical size meaning that the birds nesting there were the most concentrated in space and making it more difficult for them to exit around the net; (5) CR2 was the most continually disturbed site because it was not possible there to move the net off of the bridge between drops and thus birds were more exposed to people above their colony than at other sites; and (6) CR4 was the largest bridge, where, because of its much larger physical expanse, birds had the most space to exit around, above, or below a drop net.

Each colony-specific analysis consisted of a number of groups equal to the number of years during which the colony had been visited and a number of occasions equal to the total number of unique dates on which the colony was netted across all years (Table 2). For each year-group, if the colony was visited on a given date and an individual was captured, “1” was entered in its encounter history; if the colony was visited and the bird was not caught, “0” was entered in its encounter history; and if the colony was not visited on that date that year, “.” was entered in the encounter history. By using “.” to represent days on which a site was not visited, we avoided the deflation of daily detection probabilities that would have occurred had we treated those days as ones on which an individual was missed. We used a single parameter (within the parameter index matrices) to represent all ‘.’ occasions. Only birds caught as adults were included in these analyses. We assessed goodness-of-fit and estimated over-dispersion (ĉ) using program RELEASE (which calculates an over-dispersion estimate when “.” are present in the capture histories), used the ĉ value to adjust models for over-dispersion, and ranked models via quasi-AIC values (QAIC_c_).

CJS models estimate both apparent survival (φ) and detection (*p*) probabilities. For each colony-specific analysis, we used a simplified model of apparent survival in which daily survival varied by within-year “time since capture” and was constant among years. When building models that were used to estimate within-season detection probability, we chose a reduced parameterization for apparent survival that reflected the possibility of transience (i.e. that a swallow might be caught once and never again within a season) [57]. To account for the decreased daily apparent survival of transients within the dataset, we built parameter index matrices to reflect a within-year age structure with daily survival different for the interval following an individual’s first capture in a given year versus all other intervals. The parameterization for apparent survival used in each within-year analysis consisted of two parameters: φ_(first capture)_+φ_(after first capture)_, and is referred to as φ(null).

We built the same full-detection probability model for each of the six colony-specific within-season analyses. This model included all the covariates we believed might influence daily cliff swallow detection probability. Building the same model for each site allowed us to compare the relative support for each covariate in the model by assessing whether the 95% confidence intervals associated with regression parameters overlapped zero (equivalent to *P* = 0.05 [82]). Confidence intervals for regression parameters overlapping zero indicated poor support for a covariate in the model. We chose not to use model selection for this particular component of the analysis, as we would have, inevitably, arrived at different “best models” for each colony, making comparison of individual covariates across colonies difficult.

While we were not interested in directly estimating separate daily detection probabilities for each year, we reasoned that there were two likely sources of annual variation: (1) the total number of times a site was netted (**total**; Table 1) and (2) the colony size (number of nests) at the site (**size**).

Although an effect of sex had not been supported in previous, smaller-scale mark-recapture analyses of this population [67], [68], [83], we included sex (**sex**) as a covariate in the within-year analyses. We also added an individual-specific covariate representing the relative age of the cliff swallow (**Age**), calculated, for each year-group in the analysis, as the number of years a swallow carried a band as an adult (Table 1). To explain sources of within-season variability in daily detection rates, we included two occasion-specific linear covariates in the parameterizations for daily detection. These covariates included the actual calendar date (**date**) and the number of visits that had elapsed so far that season (**visit**, Table 1). In addition, we included a categorical trap-dependence covariate that indicated whether or not a cliff swallow had been captured on a previous visit to a site (**trap**).

While these covariates were occasion specific in that each occasion received a different value, they were also group specific, as not all occasions were represented in all years. For example, because occasions were created by compiling all dates on which a colony was visited across all years it was visited, in the year 2001 the second visit to a given colony could have occurred on occasion 5, but in 2010 the second visit may have occurred on occasion 10. Under such circumstances, the colony was visited relatively “earlier” in the season during 2001 than 2010. Although **date** and **visit** tended to co-vary such that lower numbered visits tended to occur earlier in the year, this was not always the case, and thus we reasoned that models including both covariates might be supported. We did not include breeding stage of the colony (e.g., nest-building, egg-laying, nestling-feeding) as a covariate because stage varied consistently with date, with earlier dates corresponding to nest-building periods and later dates to nestling-feeding.

Following Gimenez and Choquet [9], we used the “Cormack-Jolly-Seber model with random effects” data type in RMark to add an individual random effect to the top-supported model for detection probability. We reasoned that accounting for individual random effects in detection probability could be important in within-year analyses, as we were combining swallows caught at multiple colonies in a given year into the same groups.

### Estimating Among-Years Detection and Apparent Survival

To estimate detection probabilities for cliff swallows caught at colonies using set or drop nets over the 20-year period (1991–2010), we built a CJS recaptures-only model in RMark [80], [81] consisting of a single occasion per year for a total of 20 occasions. We assessed goodness-of-fit and estimated over-dispersion (ĉ ) with program U-CARE [84], used this ĉ value to adjust models for over-dispersion, and ranked models via QAIC_c_. We recognized three groups (**g**): swallows of unknown age when first captured that were caught using set nets (SU; *n* = 96849 birds), swallows of known age when first captured that were caught using set nets (SK; *n* = 9969), and swallows of unknown age when first captured that were caught using drop nets (DU; *n* = 57512) (Table 2). There was some overlap among groups, as 20876 swallows (∼13% of all individuals included in this analysis) were caught in set nets on one occasion and drop nets on another and thus occur in multiple groups. We did not include known-age individuals caught at drop-net sites in this analysis as the sample size of such individuals was quite small compared to that of the other three groups. Swallow age was considered known only if a swallow was originally banded in its hatching year as either a nestling or a juvenile. Our dataset was composed of swallows caught at 109 different colony sites (56 set-net and 53 drop-net sites; there were no sites at which both set- and drop nets were routinely used). To keep models from becoming too complex, when constructing the encounter histories for a given group (SU, DU, or SK), we did not indicate the specific colony site where a bird was caught.

We built age-structured parameter index matrices for apparent survival so that survival varied differently for birds in the first year they were captured (for unknown-aged individuals) or their first year as an adult (for known-aged individuals) from that in any year following that of their first capture (or first year) as an adult. This model structure allowed us to account for transient individuals who were caught once and never again [57]. We then added a year-specific component to the age structure within the parameter index matrices. Although we built separate year-specific real parameters for each age, we posited that apparent survival would likely vary by year [54], [85] and used the design matrix to build an additive model with separate intercepts for years 1991–2009. Thus, the parameterization of apparent survival that was used in all models included a total of 20 β parameters: β_φ(1st year)_+β_φ(1991)_+…+β _φ(2009)_. We tested whether cliff swallow survival varied as a linear effect of age by adding a common linear trend covariate (**Age**, Table 1) for all groups and whether there were netting group-specific differences in this age effect by adding separate **Age** trends for each netting group (g*Age). Throughout the analyses presented in this paper, age is a relative measure and generally refers to the years since first capture (or first year) as an adult, group refers to method of netting (**net**), and known or unknown-aged birds (**unk**) refer to their status when first caught as an adult (Table 1).

For detection probability, we began with a parameterization similar to that for apparent survival. Although we again built separate year-specific parameter index matrices for each age, we posited that detection would vary by year and used the design matrix to build an additive model with separate intercepts for years 1992–2010. Thus, a year (**year**) structure included 19 β parameters: β*_p_*_(1992)_+…+β*_p_*_(2010)_. We used this model to investigate whether detection probabilities varied with relative cliff swallow age and, if so, whether age could be modeled as a linear trend. We then determined whether the age trend for birds of known age when first caught as an adult (SK) was similar to that of birds of unknown age when first caught as an adult (SU), as this would indicate that “years since first banding” was a legitimate proxy for cliff swallow age. We used the design matrix to model relative age with (1) linear year-specific covariates (unique trends for SU, DU and SK, i.e. g*Age); (2) linear year-specific covariates with a separate linear trend for each netting type (unique trends for set nets and drop nets, i.e. net*Age); (3) linear year-specific covariates with a separate linear trend for each initial age type (unique trends for unknown-aged swallows and known-aged swallows, i.e. unk*Age); and (4) a common linear effect of relative age for all groups (i.e. **Age**; Table 1). We also included the sex of each individual as a categorical covariate (**sex**; Table 1). We used the “Cormack-Jolly-Seber model with random effects” data-type in RMark to add an individual random effect (in this case representing a unique effect of individual by year) to the top-supported model [9]. We fixed the individual random effect for apparent survival to zero. We present the QAIC_c_ associated with the random-effects model within the same model table as the fixed-effects-only CJS models (Table 3), as the likelihoods of these models are directly comparable [86].

**Table 3:** Set of models used in a Cormack-Jolly-Seber recaptures-only analyses of cliff swallows to test hypotheses and estimate apparent survival and detection probability among years.* ^.^

Model	ΔQAIC_c_	w_i_	K	-2LogLik	QDev
(1) φ(FY+year+g*Age), *p*(year+g*Age+RE) †	0.00	1.00	50	294863.8	937.81
(2) φ(FY+year+g*Age), *p*(year+g*Age)	116.04	0.00	49	295737.2	1055.85
(3) φ(FY+year+g*Age), *p*(year+net*Age)	150.34	0.00	47	296020.7	1094.16
(4) φ(FY+year+g*Age), *p*(year+RE)	166.96	0.00	45	296173.3	1114.77
(5) φ(FY+year+g*Age), *p*(year+unk*Age)	219.55	0.00	47	296532.9	1163.37
(6) φ(FY+year+g*Age), *p*(year+Age)	292.73	0.00	45	297104.0	1240.55
(7) φ(FY+year+g*Age), *p*(year)	301.25	0.00	44	297181.8	1251.07
(8) φ(FY+year+g*Age), *p*(year+sex)	303.32	0.00	45	297182.4	40159.78

* Parameters with interactions are joined by '*', whereas parameters having parallel (additive) relationships are joined by '+'; Akaike's Information Criterion (AIC) values were corrected for over-dispersion (see Table 3), yielding quasi-AIC (QAIC_c_) values; ΔQAIC_c_ values and model weights (w_i_) were used to rank models; see Table 1 for model notations. Here “k” indicates the number of parameters in the model and “QDev” indicates the quasi-deviance of the model.

† QAIC_c_ = 39946.48 for top-ranked model.

### Estimating Within-Year Detection during Flushing

To assess the influence of flushing on set-net capture probabilities, we restricted the analysis to the two colonies (Junkyard and Whitetail) at which flushing was conducted. We combined swallows captured at both colonies into the same analysis and used a CJS recaptures-only model to estimate the influence of flushing on the daily detection probability of cliff swallows from 2008–2010, the years during which flushes were systematically recorded.

There were 3129 cliff swallows captured at Junkyard in 2008, 3964 in 2009, and 5156 in 2010; of these, 1122 birds were caught in multiple years (and thus appear in more than one group). Between 2008 and 2010, Junkyard was visited on 47 different dates (hereafter occasions), but it was not visited on all occasions in all years, and visits were not evenly spaced. There were 1318 cliff swallows captured at Whitetail in 2008, 1527 in 2009, and 1289 in 2010; of these, 479 were caught in multiple years. Only 306 individuals were caught at both Junkyard and Whitetail during these years. Between 2008 and 2010, Whitetail was visited on 25 different occasions. Whitetail and Junkyard were visited collectively on 54 unique dates during 2008–2010, and thus we constructed encounter histories consisting of 54 occasions and included six groups in this analysis (i.e. two colonies by three years each). Encounter histories were built as described above (using “1”, “0”, and “.” if the site was not visited on that date in a given year).

We used a simplified model of apparent survival in which daily survival varied by time-since-capture within the year and was constant among years. We accounted for transients as described in the previous within-year analyses. The parameterization for apparent survival consisted of: φ_(first capture)_+φ_(after first capture)_.

We used the reduced parameterization of apparent survival to build models testing the influence of flushing (**flush**) and other covariates (Table 1) on daily detection probabilities. At Junkyard, flushes were conducted on 6 occasions in 2008, 7 occasions in 2009, and 12 occasions in 2010. At Whitetail, flushes were conducted on 4 occasions in 2008, 2 occasions in 2009, and 5 occasions in 2010. We treated a flush as a non-individual, occasion-specific categorical covariate where a “1” on a given occasion indicated a flush was done and a “0” indicated no flush was done. We built a null model in which daily detection probability was described by the same covariates we used in each of the colony-specific analyses [*p*(sex+total+size+date+visit+trap)]. We compared this to models in which we added the **flush** covariate, as well as **Age**, **colony** (e.g. Whitetail or Junkyard), and the interaction between them. We assessed goodness-of-fit and estimated over-dispersion (ĉ) using program RELEASE, and used the ĉ value to adjust models for over-dispersion as described earlier. We included individual random effects on the top-supported model and interpreted parameter estimates from this model to maintain consistency with previous analyses.

## Results and Discussion

### Within-Year Capture Effects

**Figure 3:**
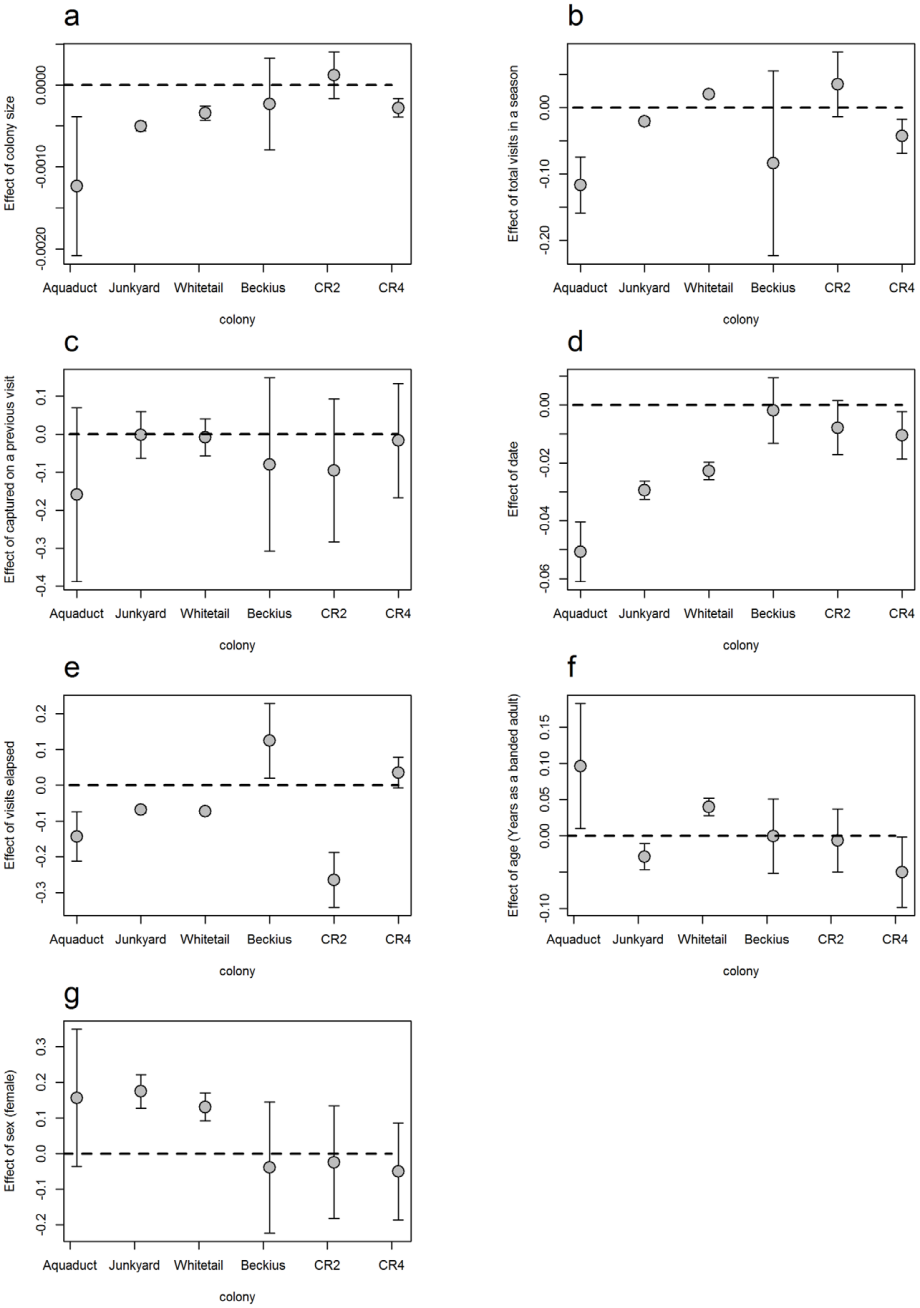
Regression coefficient estimates for cliff swallows at six colony sites, showing the effect of the following unstandardized covariates on daily detection probability: (a) colony size (size), (b) total number of visits in a season (total), (c) whether or not a swallow was captured on a previous visit (trap), (d) date, (e) visits elapsed (visit), (f) years carrying a band as an adult (Age), and (g) sex of a cliff swallow (see **Table 1**). Circles designate mean regression coefficient estimates and the vertical bars the associated 95% confidence intervals. Bars overlapping the dashed horizontal line at “0” indicate no supported relationship to detection probability; placement above the dashed line indicates a positive relationship while placement below indicates a negative relationship.

**Figure 4:**
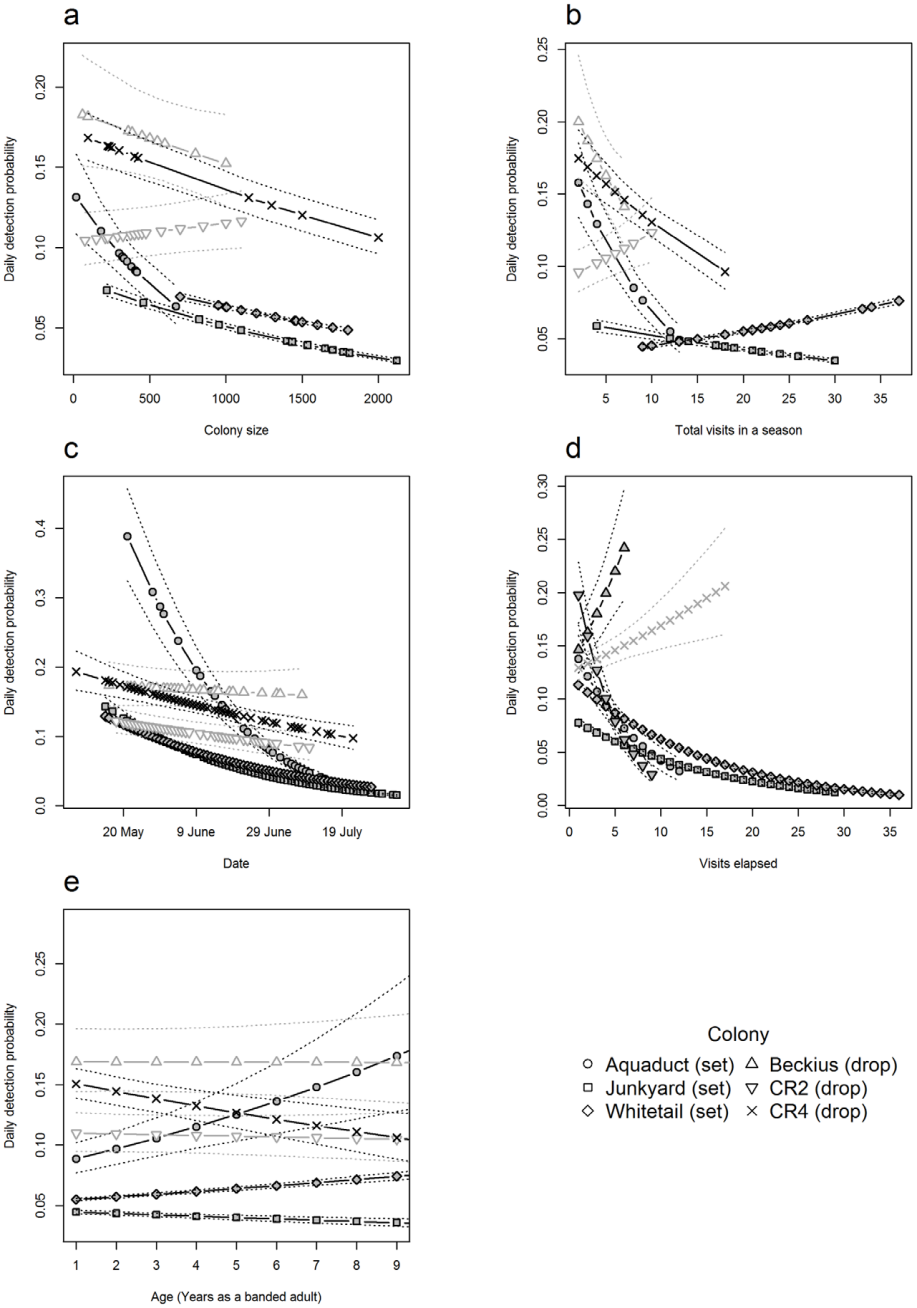
Estimates of daily detection probability (*p*) generated by the full models used in a within-year analysis for cliff swallows caught at six colony sites in relation to (a) colony size (size), (b) the total number of visits in a season (total), (c) date, (d) the number of visits elapsed (visit), and (e) the relative age of a cliff swallow (Age). Shapes connected by solid lines represent the predicted daily detection probability given the covariate value on the x-axis. When generating predicted values, all other covariates were solved at their mean value. Light gray represents unsupported relationships while dark gray represents supported ones. Dashed lines indicate 95% confidence intervals calculated using the delta method [99]. For clarity, we present daily detection probabilities for females only, as sex differences in detection probability were additive, meaning that males had a lower intercept than females but otherwise followed the same pattern.

We found that the detection probability of cliff swallows captured at set-net and drop-net colonies was associated with the colony size and the number of times, in a season, the colony was netted. Support for an effect of colony size (**size**) on daily detection probability was found for all three of the set-net sites but only one of the drop-net sites, CR4 (Figure 3a). At the four sites for which this covariate was supported, daily detection probability declined with increasing colony size (Figure 4a). Detection probability likely decreases as colony size increases because a 6-m long (4-shelf) mist net cannot hold more than about 100 swallows at once, based on the amount of mesh available for bird entanglement. Although drop nets at larger colonies were more likely to approach their capacity on each drop, swallows caught on these drops were more likely to be previously caught residents than at set net sites.

Additionally, we found support for an association between the total number of times a colony was netted in a season (**total**) and daily detection probabilities at all three set-net sites and one drop-net site, CR4 (Figure 3b). However, the direction of this effect was not consistent across colonies. At Aquaduct and Junkyard, daily detection probabilities were higher in years with fewer netting days in a season, while at Whitetail daily detection probabilities were higher in years with more netting days in a season (Figure 4b). Similar to what was observed for Aquaduct and Junkyard, daily detection probabilities at CR4 were lower in years with more netting days in a season. A negative association between the total number of colony visits and daily detection probability may reflect increased awareness of the nets (generally set in the same place each time) by cliff swallows, as suggested for other species [76], [77]. The opposite pattern, observed at Whitetail, probably reflects the use of late-season flushing to capture birds. In the early years (1991–2000), when total visits to Whitetail were highest, flushing was routinely used later in the season as researchers began experiencing declining captures (and before flushing was systematically recorded).

We did not find compelling evidence to suggest swallows demonstrated either a “trap happy” or “trap shy” response as a result of capture; we found no support for a relationship between whether or not a bird had been captured on a previous occasion (**trap**) and its detection probability (Figure 3c). Had experience in a net or being handled influenced individual behavior, we should have seen either a positive or negative relationship between daily detection probability and whether an animal was captured on a previous visit. However, the daily detection probability was not directly affected by whether an individual was caught on the previous netting visit to its colony (Figure 3c). Salewski et al. [87] similarly documented the lack of an immediate impact of mist-netting on behavior in a comparative study of stop-over stays for Palearctic migrant passerines based on recapture versus re-sighting data.

At all set-net colony sites and the drop-net site CR4, daily detection probability declined as the breeding season progressed (**date**) (Figure 4c), while controlling for the number of visits that had elapsed; at CR2, 95% confidence intervals just slightly overlapped zero, indicating possible evidence for a relationship between date and daily detection probability, while at Beckius there was no support for a relationship (Figure 3d). We believe an effect of date can be explained by cliff swallow breeding chronology. We generally began netting cliff swallows with the onset of nest-building, which tended to be relatively early in the breeding season and at a time when the birds have an incentive to frequently visit their colony to defend their nest site [67]. However, as the season progresses the birds either successfully hatch young or suffer nest failure. Either alternative leads to diminished time spent at the colony as adults concentrate on provisioning nestlings or abandon their former nesting site [79], leading to less frequent opportunities for any given individual to encounter a net even at set-net colonies that could be netted the entire season. In contrast, because drop-net colonies were netted only during a relatively narrow window of time when the birds’ presence in nests (i.e., during incubation) was most conducive to capture, our finding that calendar date had no effect on daily detection probabilities for two of these sites was not surprising.

Mist-netting effectiveness has been hypothesized to decrease as the number of days on which nets are opened increases, largely because birds are thought to become aware of the presence and location of the nets [76], [77]. Separate from (and while controlling for) the effect of date, we found that there was support for an association between the number of visits elapsed (**visit**) and daily detection probability at five of the six sites; for CR4, the 95% confidence intervals slightly overlapped zero, but were still suggestive of a positive effect (Figure 3e). However, the direction of this association was not consistent across sites. Daily detection probability declined with successive netting visit at four of the six colonies investigated (the three set-net sites and a single drop-net site, CR2), whereas it increased at the drop-net site, Beckius (Figure 4d).

Declines in detection probability with increasing site visits can be explained by an increased awareness of cliff swallows to the netting activities of the researchers; these birds apparently learn to avoid the nets over the course of the season either by being captured themselves or by watching other swallows being caught. The contradictory pattern seen at Beckius could be because there were fewer capture occasions within a year (and cumulatively) at Beckius than at any of the other sites. In addition, in one of the years, the colony at Beckius abandoned and the site was re-colonized by completely new birds within the season, which could have changed the directionality of the visit effect.

**Figure 5:**
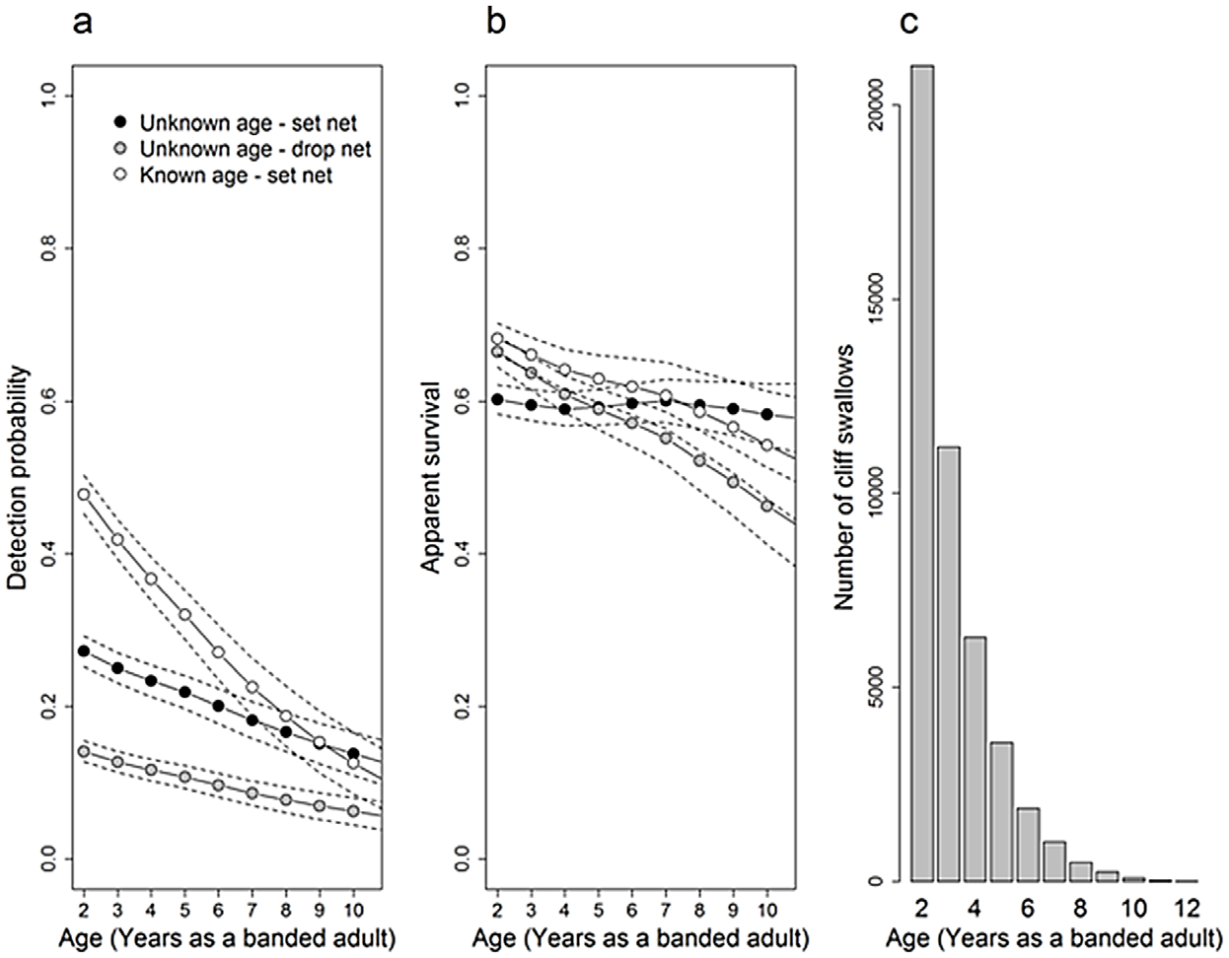
Estimates of mean annual (a) detection (*p*) and (b) apparent survival (φ) as well as the (c) age distribution of birds included in an among-years recaptures-only analysis of cliff swallows, 1991–2010. Cliff swallows included in this analysis were either of unknown age when first captured and caught in set nets (black circles), of unknown age when first captured and caught in drop nets (gray circles), or of known age when first captured and caught in set nets (white circles). Solid lines with circles indicate an age-specific mean calculated across years, dashed lines represent 95% confidence intervals estimated across all years using the delta method [99] and the top-supported model for the among-years analysis (Table 3).

While considerable work has been done to examine the efficacy of capturing different bird species using mist nets [18], [31], [76], [79] and their general safety [88], [89], to our knowledge this is the first study to compare the daily detection probability generated by passive (traditional set) mist-netting versus more active placement of nets in areas where birds simply do not have room to avoid them. Drop-netting over the side of a bridge is in many ways similar to flushing swallows into a set net, as the act of dropping the net over the side of a bridge startles the birds out of their nests and into the net. The detection probability on the second day on which netting occurred at a colony in a given year (i.e., the first opportunity for recapture) was higher at two of the drop-net colonies than at the other colonies (Figure 4d), suggesting that birds do not become familiar with drop-netting as quickly as they do with set-netting. Drop-netting thus may be a more effective method in general for catching cliff swallows or other species that can be similarly startled into nets, particularly if one has a limited time budget. However, because set-net colonies could be netted as long as any birds were resident and continuing to come and go from their nests, we visited those colonies for netting more frequently over the course of a nesting season, and ultimately we caught more birds there. As a result, the annual probability of detecting a swallow was consistently higher for swallows captured at set-net colonies than at drop-net colonies (Figure 5a). Drop-net colonies were not visited as frequently during a breeding season simply because drop-netting becomes ineffective at capturing cliff swallows once eggs hatch and parents begin feeding offspring [79]. At this point, adult swallows spend relatively little time in their nests, and drop-netting yields few captures.

**Figure 6:**
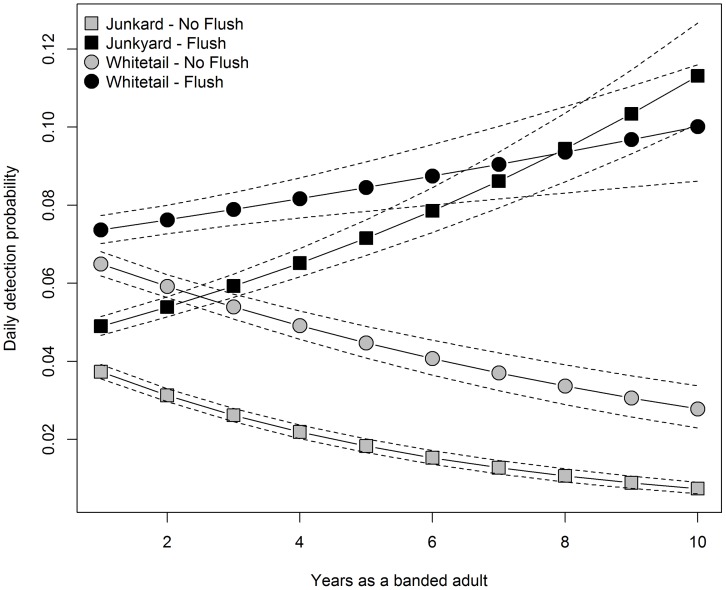
Mean estimates of daily detection probability for female cliff swallows captured at Junkyard and Whitetail (2008–2010) on days when flushing was and was not done. Dashed lines represent 95% confidence envelopes; confidence intervals and mean estimates were generated using the delta method [99]. For clarity, the effect of sex is not shown, as the relationships for each sex were the same except for a slightly lower intercept for males. All estimates were generated from the top-supported model with random effects (Table 4).

In contrast to Salewski et al. [87], who found no evidence to suggest color-banded migrants avoided mist nets in response to capture, we believe this visit-number effect (Figure 3e, Figure 4d) reflects an increase in cliff swallows’ general wariness with increased netting attempts that complements the less pronounced breeding chronology effect discussed above. Observational learning among conspecifics has been shown in various bird species [90], [91], and this kind of learning may explain net avoidance. Cliff swallows may be learning to avoid capture both by being caught themselves and by watching other swallows either get captured or avoid mist nets, or by watching humans tend a mist net. What is clear is that cliff swallows that are not caught by more passive means (i.e. a set net) are, in fact, often present in the colony. As a result, these colonies may contain individuals that are net averse and whose infrequent capture can be explained by net aversion rather than their spending disproportionally more time away from the colony. The practice of flushing was strongly associated with an increase in the daily probability of detecting a cliff swallow (Table 4; Figure 6); on average, birds were approximately twice as likely to be captured during flushes as on days when flushing was not done. At both Whitetail and Junkyard, capture probability increased with age during flushes, but capture probability declined with age during passive netting (Figure 6). The latter result suggests older swallows may have learned to avoid stationary mist nets, whereas the former result is less readily explained. One possible interpretation is that older cliff swallows respond to disturbance events more rapidly and are therefore more likely to be captured during a flush.

**Table 4:** Set of models used in a Cormack-Jolly-Seber recaptures-only analyses of cliff swallows to test hypotheses and estimate apparent survival and detection probability for the analysis of flushing.* ^.^

Model	ΔQAIC_c_	w_i_	K	-2LogLik	QDev
(1) φ(null), *p*(sex+total+size+date+visit+trap+Age*flush*colony)	0.00	0.73	14	31213.78	17340.99
(2) φ(null), *p*(sex+total+size+date+visit+trap+Age*flush*colony+RE) age*flush*sex)	2.00	0.27	15	31213.78	17340.99
(3) φ(null), *p*(sex+total+size+date+visit+trap+Age*flush)	10.45	0.00	12	31239.80	17355.44
(4) φ(null), *p*(sex+total+size+date+visit+trap+Age )	156.85	0.00	11	31506.93	17503.85

* Parameters with interactions are joined by '*', whereas parameters having parallel (additive) relationships are joined by '+'; Akaike's Information Criterion (AIC) values were corrected for over-dispersion (see Table 3), yielding quasi-AIC (QAIC_c_) values; ΔQAIC_c_ values and model weights (w_i_) were used to rank models; see Table 1 for model notations. Here “k” indicates the number of parameters in the model and “QDev” indicates the quasi-deviance of the model.

† QAIC_c_ = 17369.01 for top-ranked model.

### Transience and Apparent Survival Among-Years

If unaccounted for, the presence of transient individuals – those captured only once and never again – can reduce estimates of overall recapture probability and survival. This population of cliff swallows contains large numbers of transients; at some colonies, several hundred per day pass through the site [69]. Transient birds are most common in the early and later parts of the breeding season (C. R. Brown, unpubl. data). Early on, these individuals are generally those exploring nesting opportunities, whereas later in the season transients may include swallows that have completed reproduction for the season and are prospecting for future breeding locations [79], [92]. Consistent with past analyses [68], [69], we found evidence of transient individuals in the among-years analysis, as reflected in lower apparent annual survival in the year immediately after first capture (annual ranges in apparent survival by group: φ_SU_ = 0.30–0.54, φ_DU_ = 0.38–0.65, φ_SK_ = 0.38–0.63) than survival in subsequent years (as shown in Figure 5b). By accounting for the presence of transients in the estimates of apparent survival [57], the detection probabilities were not likely to be biased by this group of birds.

Movement among colony sites more generally may explain the differences in annual apparent survival estimates among birds of different age classes and groups (Figure 5b). For example, the drop-net colonies were among a cluster of closely spaced colonies along an irrigation canal in the study area, and for logistical reasons some of these colony sites were not included in the mark-recapture sampling. If marked birds moved to and settled permanently at these sites, they would be considered as dead/emigrated. The set-net colonies, in contrast, had fewer unmonitored neighboring sites to serve as sinks for marked birds. Bird age may also influence permanent movement away from sites [67], potentially accounting in part for the age-related differences in annual survival. More detailed studies of cliff swallow annual survival will be reported elsewhere.

### Among-Years and Within-Year Age Effects

For all groups in the among-years analysis, annual detection rates and number of individuals caught decreased as relative age increased (Figures 5a,c). This was strongly supported for all swallows captured with set nets, regardless of whether their ages were known at the time of capture or not (β*_p_*_-Age-SU_ = −0.10, 95% CI −0.21 to −0.01; β*_p_*_-Age-SK_ = −0.23, 95% CI −0.37 to −0.09; β*_p_*_-Age-DU_ = −0.11, 95% CI −0.21 to −0.01). The among-years results revealed that cliff swallows became more difficult to capture as they got older (Figure 5a). These results are broadly consistent with earlier studies that have found juvenile or yearling birds to be easier to capture in mist-nets than are adults [72], [73], [93].

However, within-season capture probabilities yielded contradictory results. An age effect on daily detection probabilities was supported at four of the six sites included in the study, while at Beckius and CR2 there was no indication that daily detection probability varied with age (Figure 3f). At Junkyard (a set-net site) and CR4 (a drop-net site), the daily detection probability of cliff swallows declined with swallow age (Figure 4e), while at Whitetail and Aquaduct (both set-net sites) daily detection probability increased with cliff swallow age (Figure 4e). We have no satisfactory explanation for these results. It is possible that at Whitetail the undocumented flushes that were done prior to 2008 may have increased the probability of detecting older birds (because flushing tends to catch older birds). This explanation is supported by the fact that, once flushing was accounted for, the detection probability of older birds declined with age at Whitetail during 2008–2010 (Figure 6).

Results of the among-years analysis suggest that there is a large proportion of older cliff swallows that are never caught in a given season. Necessarily, these older birds would also be less likely to appear in a within-year analysis as they age. Thus, we might suspect that the older swallows included in the within-year analyses are inherently different from those individuals that were never caught at older ages. Similarly, if flushing leads to the capture of birds that otherwise would not be captured in a season, we would expect a certain proportion to be caught only on days on which flushing was done. For the older ages (3+), ≥50% of all birds caught were only on days with flushes (Table 5). Additionally, at both colonies where flushing was done, daily detection probabilities were lower for older individuals on days when flushing was not done (Figure 6).

**Table 5:** Proportion of cliff swallows that were caught only on a day on which flushing was done in a given year (Flush) in relation to the total number of swallows by year (N) captured for each age.

Age	Flush	N
1	0.38	10061
2	0.35	2844
3	0.51	1378
4	0.49	728
5	0.47	484
6	0.50	383
7	0.52	218
8	0.67	150
9	0.52	79
10	0.60	40
11	0.56	9

Age represents the number of years a bird was an adult following banding.

Some studies have documented age-specific variation in detection probability as a consequence of age-related breeding propensity and philopatry (e.g., increased detection with age [12], [94]–[96]) or reproductive senescence (e.g., decreased detection with age [23], skipped breeding seasons [97]). Because we found that older individuals were more likely to be caught in flushes, and thus were present at colonies where they might otherwise not have been detected, we do not believe that breeding stage or philopatry can fully explain the decreased probability of detecting older cliff swallows. Possibly senescence could account for reduced detection with increasing age, if older birds are less active or need to forage more than younger ones and are more often absent from the colonies. Had senescence of this sort accounted for the decreased likelihood of catching older swallows, we should have also seen an age-dependent trend in recapture probability for birds caught by startling them out of their nests with drop-nets. We found that older birds did not avoid the less predictable and less avoidable drop nets more effectively than younger birds at two of the three drop-net sites (Figure 3f). Similarly, the magnitude of the relationship between relative age and annual detection probability was least pronounced for the cliff swallows caught at drop-net sites in the among-years analysis (Figure 5a). Older birds may be a subset of the population that exhibit better learning (and thus have survived), or their experience of being caught in one or more earlier years may have facilitated their awareness of the net in the current year.

### Within-Year and Among-Years Sex Effects

Female cliff swallows were slightly more likely to be recaptured than males within a season, but this effect held only at sites where stationary nets were used (Junkyard and Whitetail, Figure 3g) and sex was unrelated to recapture probability among years. Females were probably more often re-caught at set-net sites over the course of a season because they are more active during the nestling-feeding periods than males and, coming and going more, have a greater chance of encountering a net; females were 1.1 times more likely than males to be recaptured within a season (Figure 3g). We detected no effect of sex at drop-net sites for two possible reasons: these colonies could not be studied during the nestling-feeding periods, and drop-netting forces birds out of their nests and does not rely on the swallows’ normal patterns of arrival and departure from a colony. Thus, if females come and go more, we would not discern this pattern at drop-net sites.

### Conclusions

This study provides one of the most detailed explorations of recapture heterogeneity available for birds and one of the few to identify sources of that heterogeneity. A common theme was that cliff swallows had a lower probability of detection (i.e. being recaptured) as colony size and total visits in a season increased, and also had a higher probability of detection at the beginning of the breeding season than at the end. Additionally, as cliff swallows aged, their annual probability of being detected decreased. However, experienced birds’ avoidance of the net could be potentially countered by sudden disturbances that flushed them into the net before they had a chance to take circuitous routes around it. The results support the widely held assumption that birds learn to avoid nets over time but also show that modifications of traditional field methods (i.e., sudden dropping of nets from above or flushing birds into stationary nets) can reduce this source of recapture heterogeneity.

We could not investigate all potential sources of recapture heterogeneity in this population of cliff swallows; for example, some of the variation in detection may have been caused by physical features of a colony site (making it difficult to sample some parts of the colony on certain days), systematic differences in personality (e.g., extent of “boldness” [42]), changing weather conditions during capture occasions, or broader-scale weather differences among years. Inclusion of a random effect of individual by year allowed us to account for these hidden sources of detection heterogeneity while still ascertaining the strength of those covariates we chose to investigate. Our decision to examine within-season patterns in detection probability at multiple sites led to a less straightforward set of results than would have been the case had we concentrated on patterns at a single site. We are not sure which of the six sites we studied would best represent the “typical” cliff swallow colony as each poses its own set of challenges to birds [67] and researchers alike. Similarly, we did not wish to simply group birds from all colonies together because the larger numbers of cliff swallows banded and recaptured at two of the sites (Whitetail and Junkyard) would have meant that any patterns revealed would have been largely attributable to conditions at these two sites. The use of hierarchical models to control for the effect of sites as either random intercept or slope effects [66] is a promising method to deal with this situation, but currently these methods require the use of MCMC algorithms and are prohibitively slow for large datasets. Instead, we chose to draw inferences from large-scale detection patterns based on agreement among the set- and drop-net sites and whether a majority of the sites, for which an effect was statistically significant, exhibited the same directionality for a given covariate.

Mist-netting is a widely used technique for studying birds, but its limitations, brought about largely by birds’ presumed ability to learn and avoid stationary nets [76], [77], have rarely been explored. We provide strong evidence that cliff swallows avoid traditionally set mist nets at their colony sites, and such avoidance increases within a nesting season and potentially extends across multiple nesting seasons. The consequence is that the lowered detection probabilities for birds as their exposure to nets increases could affect basic demographic estimates such as age- or sex-specific survival or estimates of transition probabilities among colony sites (i.e., the observed patterns of colony choice [98]). For example, unaccounted-for age-dependence in recapture likelihood could bias age-specific survival estimates downward, suggesting senescence when none exists. Or, not accounting for transients in estimating recapture probability could mask detection of survival senescence if it does exist. Identifying and correcting for effects of age or prior capture on detection is only possible if an independent method of detecting older or previously caught individuals is available. Other studies relying solely on stationary mist-netting should consider alternative capture or re-sighting methods, account for age-dependent detection probability, and include the effects of individual heterogeneity on both survival and detection.
